# Multi-head CRF classifier for biomedical multi-class named entity recognition on Spanish clinical notes

**DOI:** 10.1093/database/baae068

**Published:** 2024-07-31

**Authors:** Richard A A Jonker, Tiago Almeida, Rui Antunes, João R Almeida, Sérgio Matos

**Affiliations:** IEETA/DETI, LASI, University of Aveiro, Campus Universitário de Santiago, Aveiro 3810-193, Portugal; IEETA/DETI, LASI, University of Aveiro, Campus Universitário de Santiago, Aveiro 3810-193, Portugal; IEETA/DETI, LASI, University of Aveiro, Campus Universitário de Santiago, Aveiro 3810-193, Portugal; IEETA/DETI, LASI, University of Aveiro, Campus Universitário de Santiago, Aveiro 3810-193, Portugal; IEETA/DETI, LASI, University of Aveiro, Campus Universitário de Santiago, Aveiro 3810-193, Portugal

## Abstract

The identification of medical concepts from clinical narratives has a large interest in the biomedical scientific community due to its importance in treatment improvements or drug development research. Biomedical named entity recognition (NER) in clinical texts is crucial for automated information extraction, facilitating patient record analysis, drug development, and medical research. Traditional approaches often focus on single-class NER tasks, yet recent advancements emphasize the necessity of addressing multi-class scenarios, particularly in complex biomedical domains. This paper proposes a strategy to integrate a multi-head conditional random field (CRF) classifier for multi-class NER in Spanish clinical documents. Our methodology overcomes overlapping entity instances of different types, a common challenge in traditional NER methodologies, by using a multi-head CRF model. This architecture enhances computational efficiency and ensures scalability for multi-class NER tasks, maintaining high performance. By combining four diverse datasets, SympTEMIST, MedProcNER, DisTEMIST, and PharmaCoNER, we expand the scope of NER to encompass five classes: symptoms, procedures, diseases, chemicals, and proteins. To the best of our knowledge, these datasets combined create the largest Spanish multi-class dataset focusing on biomedical entity recognition and linking for clinical notes, which is important to train a biomedical model in Spanish. We also provide entity linking to the multi-lingual Systematized Nomenclature of Medicine Clinical Terms (SNOMED CT) vocabulary, with the eventual goal of performing biomedical relation extraction. Through experimentation and evaluation of Spanish clinical documents, our strategy provides competitive results against single-class NER models. For NER, our system achieves a combined micro-averaged F1-score of 78.73, with clinical mentions normalized to SNOMED CT with an end-to-end F1-score of 54.51. The code to run our system is publicly available at https://github.com/ieeta-pt/Multi-Head-CRF.

**Database URL**: https://github.com/ieeta-pt/Multi-Head-CRF

## Introduction

The progress in technology has proved fruitful for the medical field throughout the years, which enhanced the quality of life for the general population. This fostered improvements in disease prevention, diagnosis, and treatment, and it can assist health professionals in performing their tasks such as clinical decision-making and patient follow-up. Unstructured data (such as free text) is typically present in clinical notes, e.g. in clinical appointment reports or patient discharge reports. Since the free text is written in natural language, it overcomes the limitations of structured information providing a flexible convoy to document complete descriptions of the patient’s health status. This is a common practice that occurs in almost every health institution, independently of the country or language.

Biomedical named entity recognition (NER) plays an important role in the information extraction from clinical texts, enabling automated analysis of patient records, and in turn supports drug development and medical research. While traditional approaches have primarily focused on single-class NER tasks, recent advancements have underscored the necessity of addressing multi-class scenarios, especially in complex biomedical domains [1]. This is an important task for this field since it pushes forward the scientific community toward an eventual goal of finding biomedical associations, such as identifying diseases given various symptoms.

In the context of the BioCreative VIII Track 2 challenge [2], which originally tackled single-class NER for symptom detection in Spanish clinical notes, we extend the task to a multi-class framework. By merging four distinct datasets, namely SympTEMIST, MedProcNER [3], DisTEMIST [4], and PharmaCoNER [5], we expand the scope of NER to encompass five classes—symptoms, medical procedures, diseases, chemicals, and proteins—considering also that each one of these should be normalized to the SNOMED CT (Systematized Nomenclature of Medicine Clinical Terms) standard [6]. Notably, these classes exhibit overlapping entities, posing a challenge for traditional NER methodologies. In common methodologies, the best solution would likely be to train several classifiers, each one of which may have the task of classifying a single entity class. However, this strategy is not scalable since it requires the training of many models, increasing both the training time and inference overhead [7–9]. Another strategy would be to use methods for handling complex named entity (NE) mentions such as nested NE mentions, overlapping NE mentions, and discontinuous NE mentions [10].

In response to the challenges posed by traditional methodologies in natural language processing (NLP), we present an innovative architecture, the multi-head conditional random field (CRF). It combines the performance of individual classifiers, with the efficiency of a single classifier. By introducing multiple classification heads on top of a shared RoBERTa based transformer model [11], we enable the classification of various classes in a unified model, ensuring scalability for multi-class NER tasks. This architecture enhances computational efficiency and it also overcomes the limitations of traditional approaches by seamlessly integrating multiple classification heads, allowing for the accommodation of an arbitrary number of entity classes while maintaining high performance.

In summary, our research aims to advance the field of multi-class biomedical NER and linking, and also to set the groundwork for more complex tasks that follow, such as extracting relationships between entities. Our main contributions in this work are the following:

A novel multi-head CRF model boasting the same performance of individual classifiers while being more scalable and faster to train and providing competitive results in entity recognition (https://github.com/ieeta-pt/Multi-Head-CRF).

- A multi-class NER model trained over a unified dataset containing 45 167 entities of five classes—symptoms, medical procedures, diseases, chemicals, and proteins—showing competitive baseline results.

## Background

In the clinical context, NER and linking serve the critical function of extracting organized data from extensive collections of unstructured clinical records. This task involves: (i) detecting essential biomedical elements specific to health care, including diseases, symptoms, therapies, medications, procedures, or other patient details; and (ii) linking these to standard clinical terminologies such as Unified Medical Language System (UMLS) [12]. The identification of these clinical concepts in the text aims to help downstream tasks, such as relation extraction and health record summarization. It can provide useful highlights when physicians are reading a patient’s clinical history [13, 14]. Most of the research in clinical NER and entity normalization has been focused on English text [15]. However, clinical notes in other languages likewise contain hidden knowledge to discover [16, 17]. In the current state of NLP, most of the work being done utilizes BERT [18] or similar transformer-based technologies.

### Previous challenges

Over the past years, there have been several initiatives to foster biomedical entity recognition. Task 1 of the ShARe/CLEF eHealth Evaluation Lab 2013 consisted of a challenge for annotation of disorder mentions in English clinical reports, where they needed to be identified and mapped to SNOMED CT through UMLS Concept Unique Identifiers [19, 20]. Following this shared task, SemEval 2014 Task 7 and SemEval 2015 Task 14 similarly promoted the development of systems for the identification and normalization of diseases and disorders but used larger test sets [21, 22]. The 2019 n2c2 Track 3 shared task focused on medical concept normalization within clinical reports, where mentions of clinical problems, treatments, and tests were linked to SNOMED CT and RxNorm terminologies [23, 24].

Social media text and scientific literature have also been the targets of entity recognition and normalization tasks. Numerous challenges have been organized by BioCreative for performing annotation of different bioconcepts in scientific literature [25–31]. Another community effort, the Bacteria Biotope task at BioNLP Open Shared Tasks 2019, focused on the identification of mentions of microorganisms, habitats, and phenotypes and their linking to reference knowledge sources (NCBI taxonomy, OntoBiotope ontology) [32]. However, all these tasks focused on resources written in English.

From the past years, the Text Mining Unit at Barcelona Supercomputing Center has been organizing a series of NLP challenges dealing with Spanish clinical text: the PharmaCoNER task consisted of identifying chemical compounds and drugs [5]; CANTEMIST focused on the detection and normalization of mentions related to cancer data [33]; the DisTEMIST task promoted the development of systems for automatic detection and normalization of disease mentions [4]; MedProcNER dealt with the detection of medical procedures [3]; and SympTEMIST was conducted for identifying symptom mentions [2].

Entity recognition and linking are crucial first tasks that are relevant for future tasks, such as relation extraction. Clinical relation extraction plays a pivotal role in automatic information extraction since it can bring new insights into health complications or disease cure hypotheses. For example, new associations between chemicals and diseases or symptoms and diseases, can be suggested for future research in medical facilities to improve well-being for everyone.

### Named entity recognition and linking

Some recent works tackle these two tasks in a multi-task learning setting using a neural network or transformer-based models to minimize the error propagation from the entity recognition to the linking step [34, 35]. Many challenges have been organized and different datasets have been released for several biomedical and clinical text mining tasks including NER and named entity linking (NEL) [15, 36–42]. Traditionally, entity recognition and entity linking are tackled sequentially and solved separately in a two-step pipeline:

1. *Named entity recognition* can be approached using dictionary-matching, which relies heavily on maintaining high-quality dictionaries or gazetteers [43], or framed as a sequence labeling problem where tokens are tagged as being part of an entity or not [44–46]. Different token-level tagging schemes exist for entity recognition [47] but, due to its simplicity, the BIO (Beginning, Inside, Outside) tagging format is commonly used in biomedical NER [48].
2. *Named entity linking* takes the detected named entities from the first step and attempts to map every single entity mention to unique code identifiers from a standard terminology [6, 12, 49]. The most simple strategies rely on exact or partial string matches over prebuilt dictionaries or make use of string similarity metrics [50, 51]. Frequently, entity linking systems have relied on sieve-based methodologies [24, 52–56], which employ a multi-stage pipeline where after each step entities that are not assigned codes are sent through the remaining sieve. Generally, common stages consist of finding direct matches over train data, direct matches over the knowledge base, and finally finding semantic matches using some form of textual embeddings.

CRF models have been extensively used for solving NER as a sequence labeling task [57] and, combined with transformer-based models, currently present the state-of-the-art results for recognizing concepts in clinical and biomedical texts [58, 59].

Despite the large number of works on entity recognition, many of the developed systems are unable to extract overlapping entities of the same type. Recently, researchers have identified this limitation and have started proposing alternative approaches capable of handling the labeling of sequences in a nested manner [60–66].

In 2023, Luo *et al*. proposed a novel all-in-one (AIO) tagging scheme that allows the recognition of multiple entity types at once [58]. They added special tokens between surrounding sentences to indicate which entity type it referred to (e.g. *<*Disease*>* for diseases and *<*Gene*>* for genes). However, their proposed method does not allow to predict entities with overlapping boundaries. Their system (AIONER) uses by default the PubMedBERT-CRF model [67, 68] for predicting six different biomedical entity types with a single transformer-based model [58].

PubTator 3.0 is a biomedical literature resource, freely provided by NCBI, which offers annotations for six biomedical entities and their normalization codes [69]. It uses AIONER for entity recognition and it employs different normalization tools for performing entity linking for every entity type—these include GNorm2 (genes), TaggerOne (diseases and cell lines), the NLM-Chem tagger (chemicals), and tmVar3 (genetic variants) [70–73].

Similarly, Sänger *et al*. proposed HunFlair2, a state-of-the-art transformer-based model for entity recognition and entity linking, which similarly to AIONER employs a single model that extracts entities of different types [74]. Kim *et al*. proposed BERN2, a neural biomedical NER and normalization tool, which employs a multi-task NER model utilizing multiple classification heads and a network-based entity linking model [59]. Their NER model has a shared backbone model and a separate task-specific layer (two-layer multilayer perceptron with rectified linear unit activation) for each entity type.

The use of ChatGPT and large language models (LLMs) has also drawn attention in the field of biomedicine and health. Tian *et al*. made an exhaustive study comparing different LLMs and concluded that despite having achieved great advances in text generation they only offer small advances in other text applications [75]. Also, LLMs such as GPT3 [76] pose legal and privacy risks and have the problem of fabricating information (hallucination).

## Methodology

In this section, we describe the dataset, the evaluation metrics used in this work, and all the details regarding the proposed system for entity detection and linking.

### Dataset

The dataset used in this work results from merging four distinct datasets that share a common set of documents. These datasets correspond to annotated versions of the 1000 clinical cases making up the Spanish Clinical Case Corpus [77], a collection of clinical case reports obtained from Spanish medical publications [78], encompassing a total of 16 504 sentences with an average of 16.5 sentences per clinical case. Each of these documents was verified to contain the same text. We imposed the same train/test split for all the corpora, with 750 of the 1000 documents reserved for training and 250 used for testing. During the development of work, we further split the train data and utilize a validation set of 250 documents. The majority of the entities mentioned in these documents have been normalized to SNOMED CT [6].

The corpora we utilize in this work are described below and their statistics are shown in Table 1:

**Table 1.** Datasets statistics with the number of entity mentions

Dataset	Train	Test	Total
SympTEMIST	9 091	3 102	12 193
MedProcNER	11 065	3 618	14 683
DisTEMIST	8 065	598	10 663
PharmaCoNER	4 665	1 959	7 624
* NORMALIZABLES*	3 246	1 152	4 398
* NO_NORMALIZABLES*	37	13	50
* PROTEIN*	2 253	756	3 009
* UNCLEAR*	129	38	167
Total	32 886	11 277	45 163

- SympTEMIST [2]: Designed to capture symptoms, signs, and findings within clinical narratives, SympTEMIST contributes with 12 193 annotations to the combined dataset. This was one of the challenges for BioCreative 2023. A total of 268 (161 + 107) codes were not normalized and were labeled as “NO_CODE.”
- MedProcNER [3]: It is focused on identifying medical procedures and contains a substantial annotation count of 14 683 instances. This was one of the challenges for BioASQ 2023. A total of 74 (57 + 17) codes were not normalized and were labeled as “NO_CODE.”
- DisTEMIST [4]: Targeting the identification of diseases, the DisTEMIST dataset comprises 10 663 annotations. This was one of the challenges for BioASQ 2022. Only one entity mention is not normalized in the dataset, and it is present in the training set.
- PharmaCoNER [5]: Originally structured with four distinct classes: “NORMALIZABLES,” “NO_NORMALIZABLES,” “PROTEIN,” and “UNCLEAR” entities. Notably, in our analysis, we join the “NORMALIZABLES” and “NO_NORMALIZABLES” classes into a unified class, “CHEMICAL,” corresponding to chemicals. “NO_NORMALIZABLES” class only contains 50 entities compared to the 4398 entities present in the “NORMALIZABLES” class. Furthermore, the “UNCLEAR” class was not evaluated in the competition, and therefore we do not use it within our dataset. This was one of the tasks of the BioNLP-OST 2019/EMNLP-IJCNLP workshop. The entities dataset contains a total of 277 (205 + 72) codes that were not normalizable to SNOMED CT. Some codes correspond to the ChEBI chemicals database [79], which were considered as not normalizable. Since this dataset follows a different train/test document split, we applied the same split as in the three other corpora to achieve consistency.

Our merged Spanish dataset contains the following entity classes:

- *SYMPTOM*: taken from SympTEMIST, corresponding to symptoms.
- *PROCEDURE*: taken from MedProcNER, corresponding to medical procedures.
- *DISEASE*: taken from DisTEMIST, corresponding to diseases.
- *PROTEIN*: taken from the PROTEIN class from PharmaCoNER, representing proteins.
- *CHEMICAL*: taken from the cumulation of “NORMALIZABLES” and “NO_NORMALIZABLES” from PharmaCoNER, representing chemicals.

**Figure 1.**
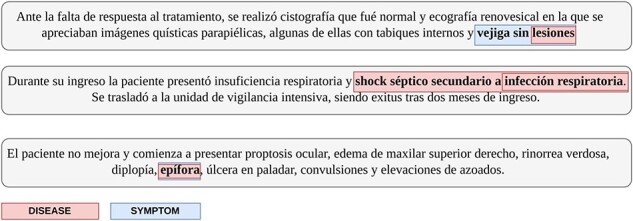
The types of overlapping entity annotations present in the dataset. Snippet 1 represents an entity of one type annotated within the span of an entity of another type; Snippet 2 represents two annotations of the same type which overlap; Snippet 3 represents one entity mention that is labeled as two different classes. Our solution is able to solve the problems that arise from the cases in Snippets 1 and 3.

While our approach is able to negate the effect of interclass overlapping entities, intraclass overlapping remains a concern. Interclass overlapping occurs when the spans of entities of two different classes overlap, whereas intraclass overlapping occurs between entities of the same class. These two types of overlap are illustrated in Fig. 1. A total of 1070 entity annotations exhibit intraclass overlap, predominantly originating from the MedProcNER and DisTEMIST datasets. An overview of this information is seen in Table 2. In our methodology, we merge these entities and train our models using the longest span of the entities.

**Table 2.** Total number of overlapping entities within the datasets

Subset	Train	Test	Total
SympTEMIST	57	39	96
MedProcNER	418	143	561
DisTEMIST	323	90	413
PharmaCoNER	0	0	0
Total	798	272	1 070

Regarding entity linking, the corpora we utilize contain entities normalized to SNOMED CT. SNOMED CT provides a standardized way of representing clinically relevant information across various health-care settings, including clinical documentation, electronic health records, decision support systems, and health data analysis. It encompasses a vast collection of structured clinical concepts, organized hierarchically, with each concept assigned a unique code. This hierarchical structure allows for precise and granular representation of medical concepts, facilitating interoperability and semantic consistency in health information exchange. SNOMED CT plays a crucial role in enhancing the accuracy, efficiency, and interoperability of health-care systems, ultimately contributing to improved patient care, clinical decision-making, and health outcomes. In this work, we employed the April 2019 SNOMED CT Spanish Edition, which contains 1 183 431 entries with 425 446 unique codes.

### Evaluation

In this work, the primary evaluation metrics are micro-averaged Precision (P), Recall (R), and F1-score, with micro-averaged F1 being the official evaluation measure for assessing and sorting the performance of participating systems in all tasks of the four challenges (SympTEMIST, MedProcNER, DisTEMIST, and PharmaCoNER). These metrics are calculated as


$$P = \frac{{TP}}{{TP + FP}},$$

$$R = \frac{{TP}}{{TP + FN}},$$

$${F_1} = 2 \times \frac{{P \times R}}{{P + R}},$$

where TP, FP, and FN correspond to the total number, summed across all classes, of True Positives, False Positives, and False Negatives, respectively.

NER followed a strict evaluation with exact match between span offsets, and similarly for entity linking, a predicted normalization code is considered a True Positive if it matches exactly against the gold standard annotation.

Table 3 presents, for every task, additional information regarding the official evaluation performed during the challenges. We note that the PharmaCoNER challenge did not include an Entity Linking task, considering instead a Concept Indexing task where for each document a set of related SNOMED CT codes had to be retrieved. Nonetheless, the original PharmaCoNER dataset contains SNOMED CT normalization codes for the large part of annotated entities which allowed us, in our work, to evaluate the NEL task for this dataset. In the case of SympTEMIST, entity linking was carried out considering that participants had access to the gold standard entity annotations, for which they had to provide the respective normalization codes. In the other two challenges, MedProcNER and DisTEMIST, entity linking was conducted in an end-to-end fashion where normalization codes were attributed to entity mentions predicted by the NER module. Moreover, some of the entities annotated within these three datasets are associated with more than one code, which are concatenated with the symbol “+,” and these are known as “composite mentions.” To evaluate these particular “composite mentions,” all the normalization codes need to match to be considered a correct prediction (True Positive). In the SympTEMIST challenge, contrarily to MedProcNER and DisTEMIST, “composite mentions” were not considered in the entity linking evaluation. Finally, the datasets contain a considerable number of entities for which an annotated normalization code is not available and which were not considered for evaluation.

**Table 3.** Notes regarding the official evaluation in the four text mining challengesThe NER task was evaluated considering exact span matches (strict evaluation). In SympTEMIST, MedProcNER, and DisTEMIST a gazetteer with a subset of SNOMED CT terms was built, by the shared task organizers, to facilitate the Entity Linking task and only codes belonging to this gazetteer were considered for evaluation

Challenge/dataset (year)	Tasks	Additional evaluation notes
SympTEMIST^a^ (2023)	NER	
	NEL	Evaluation was carried out considering gold standard entity mentions. Composite mentions were not included in the evaluation.
MedProcNER^b^ (2023)	NER	
	NEL	Entity linking was evaluated in an end-to-end fashion. Composite mentions, which are associated with more than one code, were considered for evaluation.
DisTEMIST^c^ (2022)	NER	
	NEL	End-to-end evaluation was considered with entity mentions predicted by the NER model. Composite mentions, which are associated with more than one code, were considered for evaluation.
PharmaCoNER^d^ (2019)	NER	Entity mentions of “UNCLEAR” class were not considered for evaluation. The original dataset used a different test split although it contained the same number of documents (250).

The NER task was evaluated considering exact span matches (strict evaluation). In SympTEMIST, MedProcNER, and DisTEMIST a gazetteer with a subset of SNOMED CT terms was built, by the shared task organizers, to facilitate the entity linking task and only codes belonging to this gazetteer were considered for evaluation.

a https://temu.bsc.es/symptemist/.

b https://temu.bsc.es/medprocner/ and https://github.com/TeMU-BSC/medprocner_evaluation_library.

c https://temu.bsc.es/distemist/ and https://github.com/TeMU-BSC/distemist_evaluation_library.

d https://temu.bsc.es/pharmaconer/ and https://github.com/PlanTL-SANIDAD/PharmaCoNER-Evaluation-Script.

Since slightly different NEL evaluation approaches were followed in the three challenges, we implemented our own evaluation script to facilitate model development. Therefore, we acknowledge our NEL results may not be directly comparable to other works. For simplicity, and in accordance with the most recent challenge SympTEMIST, all the results presented do not consider the evaluation of “composite mentions.” Additionally, contrarily to the official NEL evaluation in SympTEMIST, MedProcNER, and DisTEMIST, entities with normalization codes not belonging to the respective gazetteers were also considered in our evaluation.

### System

**Figure 2.**
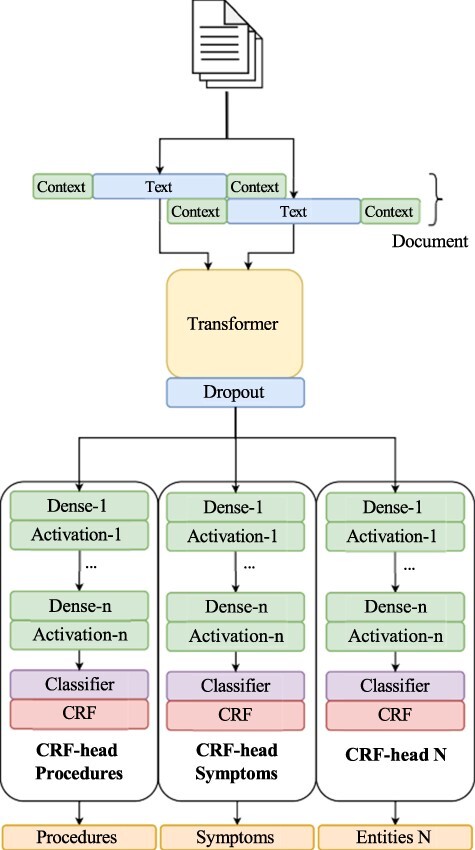
Overview of our NER pipeline, showcasing our multi-head CRF architecture.

The system architecture consists of several CRF classifier heads, allowing the model to achieve the performance of several individual classifiers while having reduced overhead. This architecture is built upon our initial submission to the BioCreative VIII track 2 competition, which was a single-class NER challenge. The original strategy used to solve the entity recognition problem framed it as a sequence labeling task, wherein tokens are classified as part of an entity or not. To facilitate this, we adopted the BIO tagging scheme. Our models are rooted in our prior work [80, 81], which leverages a transformer architecture incorporating a masked CRF as the classification layer (Fig. 2). In this work, we utilize a Spanish RoBERTa model (https://huggingface.co/lcampillos/roberta-es-clinical-trials-ner) as our transformer base, which we established to be optimal in previous works [81, 82]. Additionally, we integrate data augmentation during training. This model comprises three essential components: a transformer-based model trained in the Spanish language, an encoder layer, and a classification head.

Building upon this architecture, we extend it to use multiple CRF classifier heads, all using the same underlying transformer. The CRF layer is formulated as follows:


(1) $$P\left( {y{\mathrm{|}}x} \right) = \frac{1}{{Z\left( x \right)}}\exp \left( {\mathop \sum \limits_{i = 1}^N {f_u}\left( {{y_i},x;{\theta _u}} \right) + \mathop \sum \limits_{i = 2}^N {f_t}\left( {{y_i},{y_{i - 1}};{\theta _t}} \right)} \right),$$

where, *f_u_* is the unary function, and *f_t_* represents the transition function. The unary function computes a score for each label assigned to token *x_i_*, considering the entire sequence, while *f_t_* is the transition function, corresponding to a lookup in the transition matrix. *θ* represents the trainable parameters, and *Z*(**x**) is the partition function, acting as a normalizing factor.

The architecture employs multiple classifier heads rather than a single one. A classifier head here refers to a module comprising multiple dense layers (dense layer with activation), a classifier layer, and a CRF layer. Each of these classification heads shares the same transformer model, embedding the same text. Once the text is encoded by the transformer, it is processed by each of the several CRF heads. The model then outputs a series of labels, *C*, each corresponding to a particular entity class, *c_i_* ∈ *C*. For training this model, we used a joint loss between each class, with each head having its loss, and the model learning through the sum of these losses:


(2) $$L = \mathop \sum \limits_{i = 0}^{\left| C \right|} {L_{{c_i}}}$$

where *L_c_i* corresponds to the loss for the *i*-th classification head, which is associated with the *i*-th entity class.

As conducted in prior works [68, 80, 81], we employ a context system to overcome the limitations of the 512 context size of transformers. We split documents and incorporate a context area around each section of the document to handle longer sequences effectively. We have also employed augmentation techniques to enhance the model’s generalizability. Specifically, we use two augmentation methods: (i) random token replacement and (ii) random token replacement with unknown. In random token replacement, a random input token is replaced with a word from the vocabulary, while in augmentation with unknown, a special token “[UNK]” is used. Two hyperparameters are employed during augmentation: (i) augmentation probability, which determines the likelihood of selecting a sample for augmentation and (ii) percentage tags, which specify the proportion of tags to augment within a sample.

Regarding entity linking, we adopt the same methodology used during the competition [81]. Initially, we conduct exact matching over the training data, followed by semantic search using the multilingual SapBERT model [83]. Subsequently, cosine similarity is employed to identify the nearest matching code to that of the entity in the text ($\cos \left( {\theta } \right) = \frac{{A \cdot B}}{{\left| A \right|\left| B \right|}}$). Direct string matching over the training corpus is unnecessary as it invariably results in a 1.0 similarity score. We implement a threshold to predict non-normalizable entities, and we select the top-1 value for the code above the threshold. A threshold is used as the data contain entities that are non-normalizable.

In SympTEMIST, MedProcNER, and DisTEMIST challenges, the organizers also provided gazetteers (subsets of SNOMED CT) to help with the entity linking task by selecting only the most likely concept identifiers relevant to the entity semantic types being assessed. On the other hand, in the PharmaCoNER challenge, a gazetteer was not built to help with concept normalization and participants had to make use of the full SNOMED CT Spanish edition for the concept indexing task.

Although the approach in theory may perform worse, as there are many more clinical concepts and therefore many more candidate pairs to match with, we believe that making use of the entire vocabulary provides a more real scenario, with an eventual goal of performing NER over all possible entities presented in the SNOMED CT resource. This is the rationale for selecting the entire terminology, even though it is possible to achieve increased performance using subsets of the original vocabulary. Despite this, we also use the gazetteers as a benchmark for easier comparison to the other challenges.

## Results and discussion

In this section, we present the results obtained with the proposed system. Initially, we evaluate the performance of the model on a validation set, before using the best-performing models on a closed test set, to keep the evaluation of the model fair.

### Named entity recognition

In this work, we used a validation set of 33% of the entire training set, which corresponds to just 250 documents, leaving 500 for training. For the NER model, we considered the total F1-score, i.e. the micro-averaged F1-score over the combined dataset.

#### Validation phase

Initially, we tested the impact of context size and the number of hidden layers (Table 4). This table shows that the number of hidden layers per head plays a small role in the performance of the model, while the context size does play a part, with a context size of 32 consistently outperforming the other configurations. This result is expected, as in the few cases where the document is split, the model should benefit from using a larger context. Unfortunately, the same cannot be asserted for the number of hidden layers. With varying contexts, the optimal number of hidden layers changes, indicating that this is not an impactful parameter in the model. Nevertheless, the best-performing model uses three hidden layers and a context size of 32. Since the training time of the model was not affected by the number of hidden layers, we prioritized the training of models with three hidden layers.

**Table 4.** NER F1-scores for different contexts and hidden layers over validation data

Context	Hidden layers	F1
2	1	**76.06**
	2	75.77
	3	75.93
8	1	75.87
	2	75.78
	3	**75.90**
16	1	76.03
	2	**76.37**
	3	76.14
32	1	76.44
	2	76.38
	3	**76.52**

The best number of hidden layers is shown in bold, and the best overall system is underlined.

Following the previous test, we selected the best-performing model and conducted an experiment using various augmentation techniques, which can be seen in Table 5. Our experiments with the use of augmentation gave inconclusive results. We only improved on the results of our previous model by 0.31 percentage points, and the parameters used for augmentation vary the performance, with many combinations negatively impacting performance. The best-performing model uses random augmentation with 0.25 percentage tags and 0.5 augmentation probability. However, our findings suggest that augmentation does not have a significant impact on the models’ performance.

**Table 5.** NER F1-scores for different augmentation parameters over validation data

Aug.	Percentage tags	Aug. probability	F1
Random	0.25	0.25	76.07
		0.50	**76.83**
		0.75	76.46
	0.50	0.25	75.68
		0.50	76.01
		0.75	76.42
	0.75	0.25	75.59
		0.50	75.66
		0.75	75.85
Unknown	0.25	0.25	76.19
		0.50	76.33
		0.75	76.35
	0.50	0.25	**76.81**
		0.50	75.92
		0.75	76.29
	0.75	0.25	76.23
		0.50	76.19
		0.75	76.30

All models were trained with a context size of 32 and 3 hidden layers per head. The best result is shown in bold and underlined, the second best result is shown in bold and the third best is underlined. Aug.: augmentation.

#### Test dataset

After considering the insights gained from our validation results, we proceeded to train various models across the entire corpus (train and validation) before comparing them with those of other systems. Unless otherwise stated, the parameters used to train the models were: context size of 32, 3 hidden layers per head, random augmentation with 0.25 percentage tags, and 0.5 augmentation probability. These models were trained on the 750 training documents and evaluated on the test set containing 250 documents.

Initially focusing on our system in isolation, we aimed to assess its scalability by examining whether increasing the number of classes would affect the individual performance of each class, as shown in Table 6. The table indicates that our model maintains its performance when adding multiple classifier heads and that the number of entities considered do not significantly affect the performance. In some cases, including more entities resulted in improved performance. This may be attributed to the enhanced generalization achieved by having different heads adjust the same transformer model weights. Similarly, certain tasks exhibit relatedness, thereby contributing to a slight boost in classification.

**Table 6.** NER F1-scores for different numbers of classes over the test dataset.

Entity	2	3	4	5
Symptom	71.95	72.05	72.51	**72.57**
Procedure	**78.80**	77.94	77.85	77.90
Disease	–	77.57	77.55	**77.63**
Protein	–	–	87.33	**89.82**
Chemical	–	–	–	**92.07**
Training time	0:36:39	0:45:02	0:53:34	1:01:34

The best result is shown in bold.

Regarding the training time of the model, we observe a linear increase of approximately +8 minutes per entity class, which aligns with expectations. This time is mainly attributed to the additional CRF classifiers themselves, as varying the number of hidden layers per head did not increase the time to train the model.

The next characteristic we analyzed was the actual performance of the combined model compared to that of single-entity models trained by us on each dataset separately (using the same architecture), as well as those from the respective competitions, as displayed in Table 7. Compared to the single-class classifiers, the performance of the combined model is either superior or at most 1.25 percentage points lower. We consider this a positive outcome since it suggests that the performance of a joint classifier is comparable to that of single classifiers, while the joint classifier can be trained in significantly less time than several individual classifiers.

**Table 7.** NER comparison of individual models trained on each individual corpus versus our multi-head architecture trained on the combined corpus

Task	5 Class	1 Class	Diff.	Competition	Diff.
Symptom	**72.57**	72.53	+0.04	74.77	−2.20
Procedure	**77.90**	77.60	+0.30	79.85	−1.95
Disease	**77.63**	76.85	+0.78	77.70	−0.07
Protein	89.82	**90.97**	−1.15	88.71^a^	+1.11
Chemical	92.07	**93.32**	−1.25	94.25^a^,^b^	−2.18

The first difference corresponds to the difference between our best model trained on 5 entities against a model trained for only one entity (best is shown in bold), while the second difference corresponds to our 5-entity model versus the best in the competition (best is underlined). All models are evaluated over their corresponding test dataset. ^a^The original PharmaCoNER dataset contained a diflerent train and test split. ^b^The result for “Chemical” corresponds only to the “NORMALIZABLES” class in the original PharmaCoNER dataset.

Considering the results of the competitions, we once again observe that our performances are very close to the best results obtained from other competitors. This is encouraging for the architecture, indicating that with further fine-tuning, this strategy could potentially surpass the performance of individual classifiers altogether, allowing us to progress toward a general biomedical entity classifier. Although our results may appear slightly lagging, it is worth noting that many of these competitions involve contestants using ensemble techniques, and dedicated domain knowledge to further enhance performance, which could likely make a small difference in the performance of our system.

Finally, we trained several additional classifiers based on our previous best models to bring some additional insights into the performance of the model and the effect of the parameters. As can be seen in Table 8, our best-performing model on validation was surpassed by two models: (i) a model trained with only one hidden layer with the same augmentation; and (ii) a model trained without augmentation. This once again reiterates that the use of augmentation and the number of hidden layers did not have a significant impact on the overall performance of this model.

**Table 8.** NER F1-scores for different model parameters over test data

HLs per head	Augmentation	Percentage tags	Augmentation probability	F1
3	Random	0.25	0.50	78.73
3	Unknown	0.50	0.25	78.50
3	None	–	–	**78.89**
1	Random	0.25	0.50	**78.89**

All models are trained with a context size of 32. The best result is shown in bold. HLs: hidden layers.

### Named entity linking

We evaluated entity linking both as an end-to-end task, using our previous model, and using gold standard entities. Furthermore, we varied the source for our embeddings, namely using the gazetteers provided for the relevant competitions (none provided for Chemicals and Proteins), or the entire SNOMED CT for embeddings. As discussed earlier, it should be noted that our evaluation varies slightly from the official evaluation. Considering first the results over gold standard entities, as depicted in Table 9, we observe that when using the Gazetteer we achieve competitive results over symptoms. The remaining results are not directly comparable to the competition benchmarks. When using the entire SNOMED CT knowledge base, we do see a drop in performance, due to the increased number of codes to normalize.

**Table 9.** Entity linking scored over gold standard entities.

Threshold	Source	Chemical	Protein	Disease	Procedure	Symptom	Total
0.2	Entire SNOMED CT	79.98	84.01	**59.23**	62.77	54.06	62.98
0.4		79.81	84.01	**59.23**	**63.13**	54.06	**63.09**
0.6		**80.07**	85.87	59.11	62.45	**54.41**	**63.09**
0.8		79.90	**86.66**	52.53	56.56	49.56	58.39
1.0		75.76	76.84	34.50	47.29	41.31	47.87
0.2	Gazetteer	79.98	83.88	**61.27**	**65.23**	59.47	65.68
0.4		79.81	83.88	**61.27**	65.20	59.36	65.62
0.6		**80.07**	85.73	61.15	64.83	**59.68**	**65.72**
0.8		79.90	**86.66**	56.48	61.43	55.39	62.44
1.0		75.76	76.84	43.96	50.95	44.64	52.14
Competition	Gazetteer	-	-	56.57^a^	57.07^a^	**60.70**	

The competition scores for “Disease” and “Procedure” are from an end-to-end system. ^a^The results for “Disease” and “Procedure” during the competition were evaluated in an end-to-end setting and therefore are not directly comparable.

Our best result is shown in bold, and the best overall is shown in bold and underlined.

Table 10 presents the performance using our previous entity predictions and evaluating under an end-to-end configuration. When using a gazetteer, we achieve competitive results on procedures, and we notice a significant performance drop over symptoms when comparing to the normalization performance using gold standard entities. We further point out that our model remains competitive in diseases as an end-to-end model. In most cases, the best overall performance was obtained with a threshold of 0.6.

**Table 10.** Entity linking scored over our best-performing validation model, as an end-to-end system.

Threshold	Source	Chemical	Protein	Disease	Procedure	Symptom	Total
0.2	Entire SNOMED CT	75.78	78.08	**49.64**	53.74	44.51	54.36
0.4		**75.87**	78.08	**49.64**	**54.10**	44.51	54.49
0.6		**75.87**	**79.00**	49.60	53.80	**44.75**	**54.51**
0.8		75.78	78.60	44.87	49.87	41.35	51.18
1.0		72.49	69.78	30.56	42.34	35.34	42.81
0.2	Gazetteer	75.78	78.08	**51.46**	55.73	48.60	56.52
0.4		75.78	78.08	**51.46**	55.73	48.71	56.54
0.6		**75.87**	**79.00**	51.35	**55.95**	**48.92**	**56.72**
0.8		75.69	78.60	48.17	53.38	45.51	54.19
1.0		72.40	69.78	38.24	45.17	37.67	46.15
Competition	Gazetteer	–	–	**56.57**	**57.07**	**60.70** ^a^	

The competition score for “Symptom” uses gold standard entity annotations.

a The result for “Symptom” during the competition was evaluated directly over the gold standard entity annotations, and therefore it is not directly comparable.

Our best result is shown in bold, and the best overall is shown in bold and underlined.

### Insights

Our work was conducted on a merged multi-class entity recognition and linking dataset, facilitating the automation of entity detection and normalization across Spanish clinical texts, a crucial step toward the eventual task of automatic relation extraction. We define the NER and NEL tasks as complete if a system can identify all pertinent entities and link them to the corresponding knowledge base. This includes handling overlapping entities and entities belonging to multiple classes. Such a system sets the stage for subsequent relation extraction, allowing for the generation of accurate knowledge triples summarizing information within biomedical texts. The implementation of such a system offers numerous benefits to various stakeholders. The dataset and benchmarks used in our work were curated with these objectives in mind.

Regarding our model, we present a novel architecture that represents a significant advance toward accomplishing the NER task. While existing NER systems excel in single-class data, such as those showcased in PharmaCoNER, they are limited to performing NER on a single entity class. To address the challenges posed by multi-label overlapping entities, our work leverages state-of-the-art architectures for single-class NER and scales them up to achieve equivalent performance across various entity classes. This is achieved while reducing inference and training time by employing multiple classifiers.

We conduct an extensive architecture search, exploring various hyperparameters that may impact the performance of the model. Our findings indicate that document context segmentation enhances model performance. Additionally, we demonstrate the robustness and scalability of our model, showing that training it across multiple classes does not compromise classifier performance. In some cases, using more classes improves performance, underscoring the model’s enhanced generalizability.

We offer our end-to-end system as a baseline for future research endeavors. This system achieves an entity-linking F1-score of 54.51, which could be the benchmark for future efforts. Notably, our system uses the entire SNOMED CT resource, a deliberate choice aligned with the ultimate goal of linking to every entity type within the corpus. Our entity linking system relies on standard methodologies, demonstrating competitive performance across datasets. This comprehensive approach lays a solid foundation for advancing the field of entity recognition and linking within clinical text analysis.

For future work, we recommend a comparison to the model as an end-to-end system using the entire SNOMED CT knowledge resource, which will allow future work to drive toward a complete biomedical entity recognition and entity linking pipeline. We mainly provide alternative results using subsets of SNOMED CT to compare our system to the current state-of-the-art models.

## Conclusions

The proposed work represents a significant advance in the field of biomedical NER and linking, specifically in non-English datasets. By using a merged multi-class entity recognition and linking dataset, we address the specific challenge of detecting and normalizing overlapping entities across multiple classes in Spanish clinical texts, with normalization performed on the SNOMED CT knowledge base. To tackle this task, we propose a novel architecture, the multi-head CRF model, which combines the performance of several individual classifiers while maintaining scalability. This architecture is particularly suitable for detecting overlapping multi-class entities. Our model achieves a NER F1-score of 78.73 across the five entity classes. Utilizing the results obtained from this model, we apply traditional entity linking methodologies, resulting in competitive entity linking performance. These results can serve as benchmarks for evaluating future systems. Our end-to-end system achieves a micro-averaged F1-score of 54.51.

This work contributes significantly to the ongoing fields of NER and entity linking by describing a novel architecture for NER and benchmarks on a merged dataset for further research and development.

## References

[1] Islamaj R , Lai P-T, Wei C-H et al. The overview of the BioRED (Biomedical Relation Extraction Dataset) track at BioCreative VIII. In: *BioCreative VIII Challenge and Workshop: Curation and Evaluation in the Era of Generative Models*, New Orleans, Louisiana, USA, Zenodo, 2023.

[2] Lima-López S , Farré-Maduell E, Gasco- Sánchez L et al. Overview of SympTEMIST at BioCreative VIII: corpus, guidelines and evaluation of systems for the detection and normalization of symptoms, signs and findings from text. In: *BioCreative VIII Challenge and Workshop: Curation and Evaluation in the Era of Generative Models*, New Orleans, Louisiana, USA, Zenodo, 2023.

[3] Lima-López S , Farré-Maduell E, Gasco L et al. Overview of MedProcNER task on medical procedure detection and entity linking at BioASQ 2023. In: *CLEF 2023 Working Notes*, Thessaloniki, Greece, pp. 1–18, CEUR Workshop Proceedings. 2023.

[4] Miranda-Escalada A , Gascó L, Lima-López S et al. Overview of DisTEMIST at BioASQ: automatic detection and normalization of diseases from clinical texts: results, methods, evaluation and multilingual resources. In: *CLEF 2022 Working Notes*, Bologna, Italy, pp. 179–203. CEUR Workshop Proceedings, 2022.

[5] Gonzalez-Agirre A , Marimon M, Intxaurrondo A et al. PharmaCoNER: pharmacological substances, compounds and proteins named entity recognition track. In: *5th Workshop on BioNLP Open Shared Tasks*, Hong Kong, China, pp. 1–10. Association for Computational Linguistics, 2019.

[6] Stearns MQ , Price C, Spackman KA et al. SNOMED clinical terms: overview of the development process and project status. In: *Proceedings of the AMIA Symposium*, Washington, DC, USA, pp. 662–66. American Medical Informatics Association, 2001. PMC2243297 11825268

[7] Lee K-J , Hwang Y-S, Kim S et al. Biomedical named entity recognition using two-phase model based on SVMs. *J Biomed Informat* 2004;37:436–47. 10.1016/j.jbi.2004.08.012 15542017

[8] Galar M , Fernández A, Barrenechea E et al. An overview of ensemble methods for binary classifiers in multi-class problems: experimental study on one-vs-one and one-vs- all schemes. *Pattern Recogn* 2011;44:1761–76. 10.1016/j.patcog.2011.01.017

[9] Dong X , Qian L, Guan Y et al. A multiclass classification method based on deep learning for named entity recognition in electronic medical records. In *2016 New York Scientific Data Summit (NYSDS)*, New York, USA, pp. 1–10. IEEE, 2016.

[10] Dai X . Recognizing complex entity mentions: a review and future directions. In: Shwartz V, Tabassum J, Voigt R, Che W, de Marneffe M-C, Nissim M, (eds), *ACL 2018, Student Research Workshop*, Melbourne, Australia, pp. 37–44. Association for Computational Linguistics, 2018.

[11] Liu Y , Ott M, Goyal N et al. RoBERTa: a robustly optimized BERT pretraining approach. 2019.

[12] Bodenreider O . The unified medical language system (UMLS): integrating biomedical terminology. *Nucleic Acids Res* 2004;32:D267. 10.1093/nar/gkh061 PMC308795 14681409

[13] de la Villa M , Aparicio F, Maña MJ et al. A learning support tool with clinical cases based on concept maps and medical entity recognition. In: *2012 ACM International Conference on Intelligent User Interfaces*, Lisbon, Portugal, pp. 61–70. ACM, 2012.

[14] Pivovarov R , Elhadad N. Automated methods for the summarization of electronic health records. *J Am Med Inf Assoc* 2015;22:938–47. 10.1093/jamia/ocv032 PMC4986665 25882031

[15] French E , McInnes BT. An overview of biomedical entity linking throughout the years. *J Biomed Informat* 2023;137:104252. 10.1016/j.jbi.2022.104252 PMC9845184 36464228

[16] Pérez A , Weegar R, Casillas A et al. Semi- supervised medical entity recognition: a study on Spanish and Swedish clinical corpora. *J Biomed Informat* 2017;71:16–30. 10.1016/j.jbi.2017.05.009 28526460

[17] Weegar R , Pérez A, Casillas A et al. Recent advances in Swedish and Spanish medical entity recognition in clinical texts using deep neural approaches. *BMC Med Inf Decis Making* 2019;19:274. 10.1186/s12911-019-0981-y PMC6927099 31865900

[18] Devlin J Chang M-W Lee K et al. BERT: pre-training of deep bidirectional transformers for language understanding. In: Burstein J, Doran C, Solorio T, (eds.), *Proceedings of the 2019 Conference of the North American Chapter of the Association for Computational Linguistics: Human Language Technologies, Volume 1 (Long and Short Papers)*, Minneapolis, Minnesota, pp. 4171–86.Association for Computational Linguistics, 2019.

[19] Pradhan S , Elhadad N, South BR et al. Task 1: ShARe/CLEF eHealth evaluation lab 2013. In: *CLEF 2013 Working Notes*, Valencia, Spain, CEUR Workshop Proceedings, 2013.

[20] Suominen H Salanterä S Velupillai S et al. Overview of the ShARe/CLEF eHealth evaluation lab 2013. In: Forner P, Müller H, Paredes R, Rosso P, Stein B, (eds.), *CLEF 2013: Information Access Evaluation. Multilinguality, Multimodality, and Visualization*, pp. 212–31. Valencia, Spain: Springer Nature, 2013.

[21] Pradhan S , Elhadad N, Chapman W et al. SemEval-2014 Task 7: analysis of clinical text. In: *8th International Workshop on Semantic Evaluation (SemEval 2014)*, Dublin, Ireland, pp. 54–62. Association for Computational Linguistics, 2014.

[22] Elhadad N , Pradhan S, Gorman S et al. SemEval-2015 Task 14: analysis of clinical text. In: *9th International Workshop on Semantic Evaluation (SemEval 2015)*, Denver, Colorado, USA, pp. 303–10. Association for Computational Linguistics, 2015.

[23] Luo Y-F , Henry S, Wang Y et al. The 2019 n2c2/UMass Lowell shared task on clinical concept normalization. *J Am Med Inf Assoc* 2020;27:1529–e1. 10.1093/jamia/ocaa106 PMC7647359 32968800

[24] Luo Y-F , Sun W, Rumshisky A. MCN: a comprehensive corpus for medical concept normalization. *J Biomed Informat* 2019;92:103132. 10.1016/j.jbi.2019.103132 30802545

[25] Hirschman L , Colosimo M, Morgan A et al. Overview of BioCreAtIvE task 1B: normalized gene lists. *BMC Bioinf* 2005;6:S11. 10.1186/1471-2105-6-S1-S11 PMC1869004 15960823

[26] Morgan AA , Lu Z, Wang X et al. Overview of BioCreative II gene normalization. *Genome Biol* 2008;9:S3. 10.1186/gb-2008-9-s2-s3 PMC2559987 18834494

[27] Zhiyong L , Kao H-Y, Wei C-H et al. The gene normalization task in BioCreative III. *BMC Bioinf* 2011;12:S2. 10.1186/1471-2105-12-S8-S2 PMC3269937 22151901

[28] Jiao L , Sun Y, Johnson RJ et al. BioCreative V CDR task corpus: a resource for chemical disease relation extraction. *Database* 2016;2016:baw068. 10.1093/database/baw068 PMC4860626 27161011

[29] Arighi C , Hirschman L, Lemberger T et al. Bio-ID track overview. In: *BioCreative VI Workshop*, pp. 14–19. Bethesda, Maryland, USA, 2017.

[30] Leaman R , Islamaj R, and Zhiyong L. The overview of the NLM-Chem BioCreative VII track: full-text chemical identification and indexing in PubMed articles. In: *BioCreative VII Challenge Evaluation Workshop*, Virtual Event, pp. 108–13, 2021.

[31] Leaman R , Islamaj R, Adams V et al. Chemical identification and indexing in full-text articles: an overview of the NLM-Chem track at BioCreative VII. *Database* 2023;2023:baad005. 10.1093/database/baad005 PMC9991492 36882099

[32] Bossy R , Deléger L, Chaix E et al. Bacteria Biotope at BioNLP Open Shared Tasks 2019. In *5th Workshop on BioNLP Open Shared Tasks*, Hong Kong, China, pp. 121–31. Association for Computational Linguistics, 2019.

[33] Miranda-Escalada A , Farré E, Krallinger M. Named entity recognition, concept normalization and clinical coding: overview of the Cantemist track for cancer text mining in Spanish, corpus, guidelines, methods and results. In: *Iberian Languages Evaluation Forum (IberLEF 2020) Co-located with 36th Conference of the Spanish Society for Natural Language Processing (SEPLN 2020)*, Málaga, Spain, pp. 303–23. CEUR Workshop Proceedings, 2020.

[34] Zhao S , Liu T, Zhao S et al. A neural multi-task learning framework to jointly model medical named entity recognition and normalization. In: *Thirty-Third AAAI Conference on Artificial Intelligence*, Vol. 33, Honolulu, Hawaii, USA, pp. 817–24. Association for the Advancement of Artificial Intelligence, 2019.

[35] Zhou B , Cai X, Zhang Y et al. An end-to-end progressive multi-task learning framework for medical named entity recognition and normalization. In: *59th Annual Meeting of the Association for Computational Linguistics and the 11th International Joint Conference on Natural Language Processing (Volume 1: Long Papers)*, Virtual Event, August 1-6, pp. 6214–24. Association for Computational Linguistics, 2021.

[36] Simpson MS , and Demner-Fushman D. Biomedical text mining: a survey of recent progress. In: Aggarwal CC and Zhai C(eds.), *Mining Text Data*. New York, NY: Springer, 2012, 465–517.

[37] Jensen PB , Jensen LJ, Brunak S. Mining electronic health records: towards better research applications and clinical care. *Nat Rev Genet* 2012;13:395. 10.1038/nrg3208 22549152

[38] Huang C-C , Zhiyong L. Community challenges in biomedical text mining over 10 years: success, failure and the future. *Briefings Bioinf* 2016;17:132–44. 10.1093/bib/bbv024 PMC4719069 25935162

[39] Huang M-S , Lai P-T, Lin P-Y et al. Biomedical named entity recognition and linking datasets: survey and our recent development. *Briefings Bioinf* 2020;21:2219–38. 10.1093/bib/bbaa054 32602538

[40] Vashishth S , Newman-Griffis D, Joshi R et al. Improving broad-coverage medical entity linking with semantic type prediction and large-scale datasets. *J Biomed Informat* 2021;121:103880. 10.1016/j.jbi.2021.103880 PMC8952339 34390853

[41] Song B , Fen L, Liu Y et al. Deep learning methods for biomedical named entity recognition: a survey and qualitative comparison. *Briefings Bioinf* 2021;22:bbab282. 10.1093/bib/bbab282 34308472

[42] Jehangir B , Radhakrishnan S, Agarwal R. A survey on named entity recognition — datasets, tools, and methodologies. *Nat Lang Process J* 2023;3:100017. 10.1016/j.nlp.2023.100017

[43] Mikheev A Moens M Grover C . Named entity recognition without gazetteers. In: Thompson HS, Lascarides A, (eds.), *Ninth Conference of the European Chapter of the Association for Computational Linguistics*, Bergen, Norway, pp. 1–8, Association for Computational Linguistics. 1999.

[44] Zhou G Jian S . Named entity recognition using an HMM-based chunk tagger. In: Isabelle P, Charniak E, Lin D, (eds.), *40th Annual Meeting of the Association for Computational Linguistics*, Philadelphia, Pennsylvania, USA, pp. 473–80. Association for Computational Linguistics, 2002.

[45] Florian R , Ittycheriah A, Jing H et al. Named entity recognition through classifier combination. In: *Seventh Conference on Natural Language Learning at HLT-NAACL 2003*, Edmonton, Canada, pp. 168–71. 2003.

[46] Lample G , Ballesteros M, Subramanian S et al. Neural architectures for named entity recognition. In: *Proceedings of the 2016 Conference of the North American Chapter of the Association for Computational Linguistics: Human Language Technologies*, San Diego, CA, June, pp. 260–70. 2016.

[47] Alshammari N , Alanazi S. The impact of using different annotation schemes on named entity recognition. *Egypt Inform J* 2021;22:295–302. 10.1016/j.eij.2020.10.004

[48] Habibi M , Weber L, Neves M et al. Deep learning with word embeddings improves biomedical named entity recognition. *Bioinformatics* 2017;33:i37–i48. 10.1093/bioinformatics/btx228 28881963 PMC5870729

[49] Brown GR , Hem V, Katz KS et al. Gene: a gene-centered information resource at ncbi. *Nucleic Acids Res* 2015;43:D36–D42. 10.1093/nar/gku1055 25355515 PMC4383897

[50] Savova GK , Masanz JJ, Ogren PV et al. Mayo clinical Text Analysis and Knowledge Extraction System (cTAKES): architecture, component evaluation and applications. *J Am Med Inf Assoc* 2010;17:507–13. 10.1136/jamia.2009.001560 PMC2995668 20819853

[51] Rao D McNamee P , and Dredze M. Entity linking: finding extracted entities in a knowledge base. In: Poibeau T, Saggion H, Piskorski J and Yangarber R (eds.), *Multi-source, Multilingual Information Extraction and Summarization*. New York, NY: Springer, 2012, 93–115.

[52] D’Souza J , Vincent N. Sieve-based entity linking for the biomedical domain. In: *53rd Annual Meeting of the Association for Computational Linguistics and the 7th International Joint Conference on Natural Language Processing (Volume 2: Short Papers)*, Beijing, China, pp. 297–302. Association for Computational Linguistics, 2015.

[53] Jonnagaddala J , Rose Jue T, Chang N-W et al. Improving the dictionary lookup approach for disease normalization using enhanced dictionary and query expansion. *Database* 2016;2016:baw112. 10.1093/database/baw112 PMC4976299 27504009

[54] Wang Y , Hur B, Verspoor K et al. A multi-pass sieve for clinical concept normalization. *Trait Autom Des Lang* 2020;61:41–65.

[55] Dongfang X , Gopale M, Zhang J et al. Unified Medical Language System resources improve sieve-based generation and Bidirectional Encoder Representations from Transformers (BERT)–based ranking for concept normalization. *J Am Med Inf Assoc* 2020;27:1510–19. 10.1093/jamia/ocaa080 PMC7566510 32719838

[56] Dongfang X , and Bethard S. Triplet-trained vector space and sieve-based search improve biomedical concept normalization. In: *20th Workshop on Biomedical Language Processing*, Online, pp. 11–22. Association for Computational Linguistics, 2021. 10.18653/v1/2021.bionlp-1.2

[57] Lafferty JD , McCallum A, Pereira FCN. Conditional random fields: probabilistic models for segmenting and labeling sequence data. In: *Eighteenth International Conference on Machine Learning*, Williamstown, Massachusetts, USA, pp. 282–89. Morgan Kaufmann Publishers Inc, 2001.

[58] Luo L , Wei C-H, Lai P-T et al. AIONER: all-in-one scheme-based biomedical named entity recognition using deep learning. *Bioinformatics* 2023;39:btad310. 10.1093/bioinformatics/btad310 PMC10212279 37171899

[59] Sung M , Jeong M, Choi Y et al. BERN2: an advanced neural biomedical named entity recognition and normalization tool. *Bioinformatics* 2022;38:4837–39. 10.1093/bioinformatics/btac598 36053172 PMC9563680

[60] Wang B Wei L Wang Y et al. A neural transition-based model for nested mention recognition. In: Riloff E, Chiang D, Hockenmaier J, Tsujii J, (eds.), *Proceedings of the 2018 Conference on Empirical Methods in Natural Language Processing*, Brussels, Belgium, pp. 1011–17. Association for Computational Linguistics, 2018.

[61] Golam Sohrab M , Miwa M. Deep exhaustive model for nested named entity recognition. In: *2018 Conference on Empirical Methods in Natural Language Processing*, Brussels, Belgium, pp. 2843–49. Association for Computational Linguistics, 2018.

[62] Meizhi J , Nguyen NTH, Miwa M et al. An ensemble of neural models for nested adverse drug events and medication extraction with subwords. *J Am Med Inf Assoc* 2020;27:22–30. 10.1093/jamia/ocz075 PMC6913208 31197355

[63] Meizhi J , Miwa M, Ananiadou S. A neural layered model for nested named entity recognition. In: *2018 Conference of the North American Chapter of the Association for Computational Linguistics: Human Language Technologies, Volume 1 (Long Papers)*, New Orleans, Louisiana, USA, pp. 1446–59. Association for Computational Linguistics, 2018.

[64] Fisher J , and Vlachos A. Merge and label: a novel neural network architecture for nested NER. In: *Proceedings of the 57th Annual Meeting of the Association for Computational Linguistics*, Florence, Italy, pp. 5840–50. 2019. https://doi.org/ 10.18653/v1/P19-1585

[65] Yaseen U , Gupta P, Schütze H. Linguistically informed relation extraction and neural architectures for nested named entity recognition in BioNLP-OST 2019. In: *5th Workshop on BioNLP Open Shared Tasks*, Hong Kong, China, pp. 132–42. Association for Computational Linguistics. 2019.

[66] Sun L , Fule J, Zhang K et al. Multilayer ToI detection approach for nested NER. *IEEE Access* 2019;7:186600–08. 10.1109/ACCESS.2019.2961118

[67] Gu Y , Tinn R, Cheng H et al. Domain-specific language model pretraining for biomedical natural language processing. *ACM Trans Comput Healthc* 2021;3:1–23. 10.1145/3458754

[68] Luo L , Lai P-T, Wei C-H et al. BioRED: a rich biomedical relation extraction dataset. *Briefings Bioinf* 2022;23:bbac282. 10.1093/bib/bbac282 PMC9487702 35849818

[69] Wei C-H , Allot A, Lai P-T et al. PubTator 3.0: an AI-powered literature resource for unlocking biomedical knowledge. *Nucleic Acids Research* 2024;52:W540–6. 10.1093/nar/gkae235 38572754 PMC11223843

[70] Wei C-H , Luo L, Islamaj R et al. GNorm2: an improved gene name recognition and normalization system. *Bioinformatics* 2023;39:btad599. 10.1093/bioinformatics/btad599 PMC10612401 37878810

[71] Leaman R , Zhiyong L. TaggerOne: joint named entity recognition and normalization with semi-Markov Models. *Bioinformatics* 2016;32:2839–46. 10.1093/bioinformatics/btw343 27283952 PMC5018376

[72] Islamaj R , Leaman R, Kim S et al. NLM-Chem, a new resource for chemical entity recognition in PubMed full text literature. *Scientific Data* 2021;8. 10.1038/s41597-021-00875-1 PMC7994842 33767203

[73] Wei C-H , Allot A, Riehle K et al. tmVar 3.0: an improved variant concept recognition and normalization tool. *Bioinformatics* 2022;38:4449–51. 10.1093/bioinformatics/btac537 35904569 PMC9477515

[74] Sänger M , Garda S, David Wang X et al. HunFlair2 in a cross-corpus evaluation of biomedical named entity recognition and normalization tools. 2024. 10.1093/bioinformatics/btae564 PMC11453098 39302686

[75] Tian S , Jin Q, Yeganova L et al. Opportunities and challenges for ChatGPT and large language models in biomedicine and health. *Briefings Bioinf* 2024;25:bbad493. 10.1093/bib/bbad493 PMC10762511 38168838

[76] Brown T , Mann B, Ryder N et al. Language models are few-shot learners. *Adv Neural Inf Process Syst* 2020;33:1877–901.

[77] Intxaurrondo A , Krallinger M. SPACCC. 2019.

[78] Bojo-Canales C , Melero R. Open access editorial policies of SciELO health sciences journals. *J Inf Sci* 2023;49:685–98. 10.1177/01655515211015135

[79] Hastings J , Owen G, Dekker A et al. ChEBI in 2016: improved services and an expanding collection of metabolites. *Nucleic Acids Res* 2015;44:D1214–D1219. 10.1093/nar/gkv1031 26467479 PMC4702775

[80] Almeida T , Jonker RAA, da Silva D et al. BIT.UA at Biocreative VIII track 1: a joint model for relation classification and novelty detection. In: *BioCreative VIII Challenge and Workshop: Curation and Evaluation in the era of Generative Models*, New Orleans, Louisiana, USA, Zenodo. 2023.

[81] Jonker RAA , Almeida T, Matos S et al. Team BIT.UA @ BC8 SympTEMIST Track: a two-step pipeline for discovering and normalizing clinical symptoms in Spanish. 2023.

[82] Campillos-Llanos L , Valverde-Mateos A, Capllonch-Carrión A et al. A clinical trials corpus annotated with umls© entities to enhance the access to evidence-based medicine. *BMC Med Inf Decis Making* 2021;21:1–19. 10.1186/s12911-021-01395-z PMC7898014 33618727

[83] Liu F , Shareghi E, Meng Z et al. Self-alignment pretraining for biomedical entity representations. In *2021 Conference of the North American Chapter of the Association for Computational Linguistics: Human Language Technologies* Online, pp. 4228–38. Association for Computational Linguistics. 2021.

